# Raising the pH of the Pulsing Solution Improved the Acropetal Transport of NAA and 2,4-D and Their Efficacy in Reducing Floret Bud Abscission of Red Cestrum Cut Flowers

**DOI:** 10.3389/fpls.2020.00825

**Published:** 2020-06-24

**Authors:** Bekele Abebie, Sonia Philosoph-Hadas, Joseph Riov, Moshe Huberman, Raphael Goren, Shimon Meir

**Affiliations:** ^1^Department of Postharvest Science, Agricultural Research Organization, The Volcani Center, Rishon LeZion, Israel; ^2^The Robert H. Smith Institute of Plant Sciences and Genetics in Agriculture, The Robert H. Smith Faculty of Agriculture, Food and Environment, The Hebrew University of Jerusalem, Rehovot, Israel

**Keywords:** acropetal auxin transport, *Cestrum elegans*, *CeIAA1* transcript level, cut flowers, floret bud abscission, NAA, 2, 4-D, pH

## Abstract

The use of auxins to improve the vase life of cut flowers is very limited. Previous studies demonstrated that a pulse treatment of Red Cestrum (*Cestrum elegans* Schlecht.) cut flowers with 2,4-dichlorophenoxyacetic acid (2,4-D) significantly reduced floret bud abscission, whereas 1-naphthaleneacetic acid (NAA) was ineffective. This difference resulted, at least in part, from the higher acropetal transport capability of 2,4-D compared to that of NAA. The present research focused on examining the factors affecting the acropetal transport, and hence the efficacy of the two auxins in reducing floret bud abscission of Red Cestrum cut flowers. We assumed that the differential acropetal transport capability of the two auxins results from the difference in their dissociation constants (pKa), with values of 2.75 and 4.23 for 2,4-D and NAA, respectively, which affects their pH-dependent physicochemical properties. Thus, increasing the pH of the pulsing solution above the pKa of both auxins might improve their acropetal movement. Indeed, the results of the present research show that raising the pH of the pulsing solution to pH 7.0 and above improved the efficacy of the two auxins in reducing floret bud abscission, with a higher effect on 2,4-D than that on NAA. Raising the pH of the pulsing solution decreased the adsorption and/or uptake of the two auxins by the cells adjacent to the xylem vessels, leading to an increase in their acropetal transport. The high pH of the pulsing solution increased the dissociation and hence decreased the lipophilicity of the auxin molecules, leading to improved acropetal movement. This effect was corroborated by the significant reduction in their 1-octanol/water partition coefficient (K_*OW*_) values with the increase in the pH. A significant increase in the *CeIAA1* transcript level was obtained in response to 2,4-D pulsing at pH 7.0 and 8.25 and to NAA pulsing at pH 8.25, indicating that the acropetally transported auxins were taken up by the cells under these conditions. Our data suggest that raising the pH of the pulsing solution would significantly contribute to the increased efficacy of auxins in improving the vase life of cut flowers.

## Introduction

The vase life of cut flowers is limited by the acceleration of several processes, such as senescence and abscission or wilting of their various organs after harvest (Reid and Jiang, 2012). Application of growth regulators is one of the various technologies developed to improve the vase life of cut flowers. Treatments with cytokinins and gibberellins were reported to have a positive effect on the vase life of a wide range of cut flowers, by retarding leaf senescence and/or flower senescence or abscission, and by improving flower opening (Ascough et al., 2005; Reid and Jiang, 2012). Although auxins play a major role in the control of the abscission process by rendering the abscission zone (AZ) insensitive to ethylene (Meir et al., 2015), and they were also reported to retard petal senescence (Wojciechowska et al., 2018), they are seldom being used to improve the vase life of cut flowers. There are only a very few reports on the improvement of the vase life of cut flowers by auxins, applied either by pulsing or a quick dip of the whole flowers in the treatment solution. These reports include treatments with 2,4-dichlorophenoxyacetic acid (2,4-D) in carnations (Sacalis and Nichols, 1980) and 1-naphthaleneacetic acid (NAA) in Alstroemeria (Bagheri et al., 2012). Other reports demonstrated that auxin alone was ineffective, but when applied together with cytokinins or ethylene inhibitors, the combined treatment improved the vase life of cut flowers. These reports include a combined treatment of 2,4-D and benzyladenine in daffodils (*Narcissus pseudonarcissus*) (Ballantyne, 1965), 2,4-D and silver thiosulfate (STS) in Red Cestrum (Meir et al., 1999), and NAA and aminoethoxyvinylglycine (AVG) in lisianthus (*Eustoma grandiflorum*) (Shimizu-Yumoto and Ichimura, 2010a). Based on the results of the latter report, the above authors stated that auxin treatment might have a potential to improve the vase life of cut flowers (Shimizu-Yumoto and Ichimura, 2010b).

Application of chemicals to cut flowers is usually performed by pulsing, which is the common method used by growers. However, application of auxins by this method might be ineffective, since their acropetal transport might be limited. There are two main pathways of auxin transport in plants (Petrášek and Friml, 2009). The first is carrier-regulated cell-to-cell polar transport, and the second is non-directional transport in the phloem, which is commonly related to the transport of indole-3-acetic acid (IAA) from source organs. However, the above-mentioned reports demonstrating improved vase life of cut flowers by various auxins applied by pulsing, indicate that auxins are also transported acropetally, presumably in the xylem. Acropetal transport of auxins was also demonstrated in other systems by using radiolabeled auxins, including transport of IAA and indole-3-butyric acid (IBA) in shoot sections of *Arabidopsis thaliana* (Ludwig-Müller et al., 1995), NAA in loblolly pine (*Pinus taeda* L.) cuttings (Diaz-Sala et al., 1996), and IAA and IBA in mung bean cuttings (Weisman et al., 1988). Our research group demonstrated a significant fast acropetal transport of 2,4-D in cut Red Cestrum shoot sections, whereas NAA moved mostly polarly in this system (Abebie et al., 2005, 2006).

There are acceptable insights about the factors affecting the acropetal transport of weak acids in the xylem, which are relevant to most common auxins (Trapp, 2004; Kramer, 2006). Indolic auxins and NAA have a dissociation constant (pKa) between 4 and 5. In the weak acidic xylem sap, part of the molecules of these auxins will be protonated, and hence membrane permeable. Upon entering the cells adjacent to the xylem vessels, these molecules will be dissociated in the almost neutral cytoplasm, and accumulate within the cells. This mechanism, known as ion trapping (Sterling, 1994), limits the acropetal movement of auxins in the xylem, particularly if trans-membrane efflux carriers are not present. The pKa also affects the adsorption rate of various molecules onto plant cell wall components, namely lignin (Barak et al., 1983; Trapp et al., 2001), a process that might also reduce the acropetal movement of auxins. The pKa of 2,4-D (2.75) is significantly lower than that of indolic auxins and NAA, and hence 2,4-D is expected to have a relatively higher capability of acropetal transport when applied by pulsing by the commercial acidic preservative solutions, as indeed demonstrated in our previous studies (Abebie et al., 2005, 2006). The acropetal transport of auxins in the xylem is a passive process, so that the involvement of auxin transporters in this process is indirect, and could be carried out by an effect on the ion trapping mechanism. It is a common view that the accumulation of weakly acidic auxins in cells is mainly regulated by the activity of influx and efflux carriers (Grones and Friml, 2015). However, there are indications that there is a tendency of weak acids present in the extracellular space to be trapped by adjacent cells due to dissociation in the almost neutral cellular pH (Kramer, 2006).

The transport of a molecule in the plant vascular systems is often related to the relationship between its pKa and its lipophilicity (Grimm et al., 1986; Rigitano et al., 1987). Lipophilicity of a compound is commonly expressed by its *n*-octanol/water partition coefficient (K_*OW*_), whose value is mostly determined by its pKa (Briggs et al., 1987; Chamberlain et al., 1996). The K_*OW*_ value determines the lipophilic-hydrophilic balance, which in turn determines the ease of movement across plant membranes. The importance of K_*OW*_ as an indicator of lipophilicity in biological studies has been well established (Leo et al., 1971; Leo, 2000). Organic compounds can be classified as lipophilic when the log K_*OW*_ > 0, and as hydrophilic when the log K_*OW*_ < 0 (Popp et al., 2005). According to Bertosa et al. (2003), the lipophilicity positively correlates with membrane permeability and receptor binding of auxin molecules. Although the K_*OW*_ is a good indicator for measuring lipophilicity and adsorption of a xenobiotic, there are some anomalies when it comes to hydrophilic compounds. For example, the adsorption of the hydrophilic compounds onto cuticular membranes is significantly higher than expected from their K_*OW*_ values (Kerler and Schönherr, 1988).

We previously observed that 2,4-D exhibited a high efficacy in improving the vase life of Red Cestrum cut flowers by inhibiting their floret bud abscission, whereas NAA had almost no effect (Abebie et al., 2005, 2006, 2007). Similarly, a combined treatment of STS and 2,4-D inhibited floret bud abscission in Red Cestrum cut flowers, whereas a combination of STS and NAA was ineffective (Meir et al., 1999). Based on the above mentioned observations regarding the higher acropetal transport capability of 2,4-D in Red Cestrum compared to that of NAA (Abebie et al., 2005, 2006), we assumed that the difference in the response to the above treatments resulted from the differential acropetal transport capability of the two auxins. The difference in the acropetal transport capability is undoubtedly related to different physicochemical characteristics of the two auxins, namely the significantly higher pKa of NAA (4.23) than that of 2,4-D (2.75), which affect their membrane permeability and adsorption onto plant cell wall components, and possibly also apoplastic proteins. Hence, increasing the pH of the pulsing solution well above the pKa might decrease the efficacy of the ion trapping mechanism of the applied auxins and their adsorption onto plant cell wall components, resulting in their increased acropetal transport.

The aims of the present study were: (a) to examine the effect of the pH of the pulsing solutions of NAA and 2,4-D on their differential acropetal transport in relation to their efficacy in reducing floret bud abscission in Red Cestrum cut flowers; (b) to study the effect of pH on the physicochemical properties of these auxins in relation to their differential acropetal transport capability. The data of the present study indeed confirmed our assumption. Raising the pH of the pulsing solution of the two auxins well above their pKa, with the required raise in the pH being higher for NAA than that for 2,4-D, significantly increased their acropetal transport in Red Cestrum cut flowers, resulting in reduced bud abscission. Therefore, adjusting the pH of the pulsing solution of auxins might significantly increase their efficacy in improving the vase life of cut flowers, and therefore might enable the use of mild and hence less phytotoxic auxins, such as NAA, for treating cut flowers.

## Materials and Methods

### Plant Material

**FIGURE 1:**
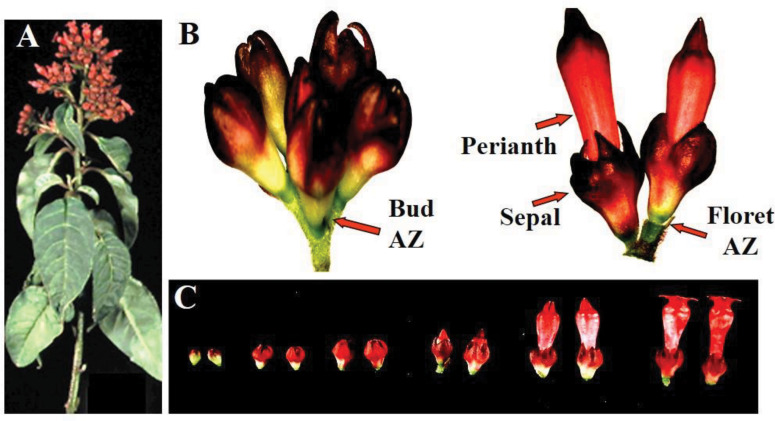
Morphology of floret developmental stages in Red Cestrum cut flowers. A typical cut flowering shoot **(A)**; definition of floral parts in an individual cluster of florets from an inflorescence head composed of florets at two developmental stages **(B)**; and classification of floret buds developmental stages **(C)**.

Red Cestrum (*Cestrum elegans* Schlecht cv. “Red Flame”) cut flowers were obtained from plants grown in a local commercial plantation. A typical cestrum cut flower shoots has an inflorescence composed of alternate racemes bearing a cluster of florets at different stages of development (Figure 1A). The florets in the upper shoot apex and the raceme apexes are chronologically older and open first, while those in the lower positions are chronologically younger and open last. Each individual inflorescence head contains also florets at different stages of development (Figures 1B,C). Generally, the experiments were performed with commercial size cut flowers bearing a few open florets, except for one experiment in which shoot segments were used.

### Radiochemicals

[1-^14^C]NAA (specific activity 344 MBq mmol^–1^) and [1-^14^C]2,4-D (specific activity 592 MBq mmol^–1^) were obtained from Sigma, United States. The purity of the radiochemicals was checked periodically by thin-layer chromatography, using silica gel GF_254_ plates developed with chloroform-ethyl acetate-formic acid (5:4:1, v/v).

### Pulsing Treatments

Cut flowers were incubated at room temperature for about 2 h until they lost 3–5% of their fresh weight (FW), in order to increase the uptake of the applied auxins. For the experiments, 30-cm-long cut flowers were used. The leaves were removed up to 12 cm from the base of the shoots, and each shoot was placed in a 50-mL Falcon tube containing 7 mL of the pulsing solution composed of 0.2 M buffer for obtaining the desired pH, and 0.2 mM of NAA or 2,4-D with or without the corresponding radiolabeled auxins as tracers. The buffers used were Na_2_HPO_4_-citrate buffer for obtaining pH levels up to 7.0, and Tris–HCl buffer for obtaining pH levels above 7.0. The cut flowers were pulsed for 24 h in an observation room maintained at 20°C, 60–70% relative humidity, and 12-h photoperiod at a light intensity of 14 μmol m^–2^ s^–1^. In one experiment, the pulsing treatment was performed for 4 h at 20°C followed by additional 20 h at 4°C. After pulsing, the lower 2-cm section of each shoot was trimmed off, and the pulsing solution was replaced with 20 mL of a disinfectant aqueous solution containing 50 μL L^–1^ sodium dichloroisocyanurate, pH 6.0 (TOG-6^®^, Gadot-Agro Ltd., Israel). The shoots were incubated in the observation room under the conditions specified above for vase life evaluation. Additional TOG-6 solution was added during vase life when necessary to replace the amount lost by transpiration.

### Monitoring Floret Bud Abscission

For monitoring floret bud abscission during vase life, individual inflorescence heads were removed from the cut flowers at different time points, placed in polyethylene bags, tapped gently, and the abscised floret buds were collected and counted. At the end of the experiment, the floret buds that did not abscise were counted, and their number was summed up to the number of abscised ones to determine the percentage of accumulated floret bud abscission during vase life.

### Autoradiography of the Acropetal Transport of NAA and 2,4-D in Cut Flowers

For determining the acropetal transport of NAA or 2,4-D by autoradiography, cut flowers were pulsed for 24 h with the standard auxin pulsing solutions containing 16.0 KBq of either [1-^14^C]NAA or [1-^14^C]2,4-D as tracers. At the end of a 24-h pulsing treatment (0 h), the lower part of each cut flower shoot was thoroughly washed with distilled water, and the cut flowers were dried off by pressing them for 1 week between 20 × 40-cm blotting papers at room temperature. The dried samples were exposed to Fujifilm Imaging Plates for 48 h, the images were then analyzed by a Fujifilm Fla-5000 phospho-imager, and processed with Photoshop version 7.0.

### Measuring the Adsorption and/or Uptake of NAA and 2,4-D by Xylem Shoot Cells

Adsorption and/or uptake of radiolabeled NAA or 2,4-D by shoot xylem sections were measured as previously described by Huberman et al. (2005) with some modifications. Samples of 200 mg of 2-3-mm-long sections of xylem tissue were pre-incubated for 5 min in 25-mL Erlenmeyer flasks containing 5 mL of 20 mM Na_2_HPO_4_-citrate buffer (for pH 3.0, 5.0, and 7.0), or 20 mM Tris–HCl buffer (for pH 9.0) to equilibrate the extracellular pH to the desired values. The above pre-incubation procedure was repeated three times. The pre-incubation buffers were removed by suction, and 2 mL of the corresponding incubation buffers containing 0.2 mM of NAA or 2,4-D and 3.6 KBq of [1-^14^C]NAA or 3.8 KBq of [1-^14^C]2,4-D as tracers were added. The sections were then incubated for 1 h at 25°C in a shaking water bath. At the end of the incubation, the incubation buffers were removed by suction, and the samples were extensively washed three times with 5 mL of the same buffer for 5 min to remove the label from the apoplast. After the final wash, the samples were blotted onto a paper towel, and then the radioactivity was extracted with 10 mL of Opti-Fluor^®^ high performance flash-point liquid scintillation cocktail for aqueous samples (Packard BioScience, United States) by shaking overnight in darkness. The extracted radioactivity was then monitored by a Liquid Scintillation Analyzer (Packard Tri-Carb 1600 TR, United States). The calculated auxin adsorption and/or uptake is expressed as nmol g^–1^ FW.

### Determination of *CeIAA1* RNA Transcript Level

Total RNA was extracted from the floret AZ following the cetyltrimethylammonium bromide (CTAB) extraction procedure, as described by Liao et al. (2004) with some modifications, following the LiCl overnight precipitation and centrifugation (20,000 × *g* for 20 min at 4°C). After a subsequent centrifugation (20,000 × *g* for 40 min at 4°C), the pellet was resuspended in 0.5 mL of sterile water and transferred to sterile Eppendorf tubes. Then, 1.8 volume of absolute ethanol and 0.1 volume of 3.0 M sodium acetate (pH 5.5) were added, and the RNA was precipitated by an overnight incubation at −20°C, pelleted by centrifugation (20,000 × *g* for 20 min at 4°C), washed twice with 75% ethanol, and resuspended in an appropriate amount of sterile water. The RNA was quantified by NanoDrop^®^ ND-1000 spectrophotometer (NanoDrop Technologies, Inc., Rockland, DE, United States), and stored at −80°C for further use. Equal amounts of total RNA (20 μg) were run on a formaldehyde agarose gel and blotted onto Hybond N^+^ membranes (Amersham Pharmacia Biotech, United States) by the standard capillary transfer methods (Ausubel et al., 1995). Membranes were hybridized with ^32^P-labeled *CeIAA1* (Gene bank accession no. DQ900819) gene specific probe amplified from the 3′UTR of the clone. The transcript level of this gene was compared by quantitative real time PCR (qRT-PCR) analysis performed as described by Abebie et al. (2007). The sequences of the forward and reverse primers used for the qRT-PCR analysis were 5′-CACCAACATATGAAGACAAGG-3′, and 5′-GCTTCAGAACCCTTCATG-3′, respectively. Two separate experiments were performed with similar results, in which the qRT-PCR reactions were performed in duplicates, thus representing overall four biological replicates.

### Determination of Physicochemical Properties of NAA and 2,4-D

The partition coefficient (K_*OW*_) values of NAA and 2,4-D were determined using the shake-flask method, as described by the Organization for Economic Cooperation and Development (OECD, 1987) guidelines for testing of chemicals. *n*-Octanol saturated with 20 mM Na_2_HPO_4_-citrate buffer (pH 3.0, 5.0, and 7.0), or 20 mM Tris–HCl buffer (pH 9.0) were used as the organic phase, and the above buffers saturated with *n*-octanol were used as the aqueous phase. The ratio of the organic phase to the aqueous phase was 1:1 (v/v), and the final concentration of NAA or 2,4-D dissolved in the aqueous phase was 2 mM. Each treatment was performed in four repetitions. Blanks were prepared in an identical manner, except that no auxin was added. The organic and the aqueous phases were allowed to reach equilibrium on a horizontal shaker for 24 h at 20°C. After equilibrium was achieved, the mixed solutions were centrifuged at 1,500 rpm for 15 min. The aqueous phase was carefully removed with a pasture pipette, and the absorbance of NAA or 2,4-D in the two phases was determined at 280 nm with a UV 2201 UV-VIS spectrophotometer against the above blanks. When required, the samples were diluted before measuring the absorbance. K_*OW*_ values were calculated from the equilibrium ratio of the auxin concentrations in the *n*-octanol and the aqueous phases, as extrapolated from standard curves.

Percent ionization was calculated by rearranging the Henderson–Hasselbalch equation at a known pH and pKa of a xenobiotic,^1^ as follows:

$$\%\text{ionization}=\frac{10^{\mathrm{pH}-\mathrm{pKa}}}{1+10^{\mathrm{pH}-\mathrm{pKa}}}\times 100$$

## Statistical Analysis

The statistical analysis was performed using JMP 5.0 software (SAS Institute). The data were analyzed using one-way ANOVA. Significant differences between treatment means were determined by Tukey’s HSD test (*P* ≤ 0.01). Experiments were repeated at least twice, and the data from one representing experiment are presented. The number of replicates in each experiment is specified in the legends of the table and figures.

## Results

### Effect of the pH of the Pulsing Solution on the Efficacy of NAA and 2,4-D in Inhibiting Floret Bud Abscission

**FIGURE 2:**
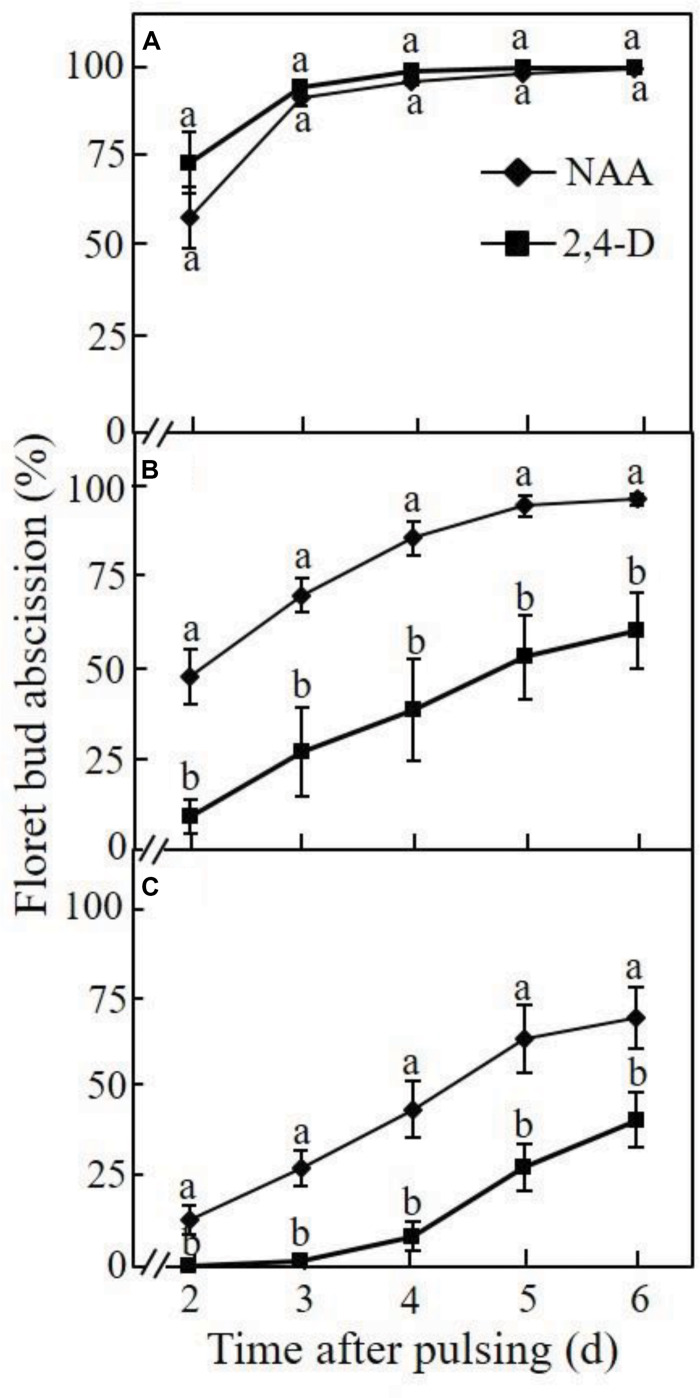
Effect of the pH of the auxin pulsing solution on the efficacy of NAA and 2,4-D to retard floret bud abscission in Red Cestrum cut flowers during vase life. The cut flowers were pulsed for 24 h at 20°C with either 0.2 mM NAA or 2,4-D at pH = pKa **(A)**, pH = 7.0 **(B)**, or pH = 8.25 **(C)** and then placed in TOG-6 solution under vase life conditions for monitoring bud abscission. The percentage of abscised floret buds was determined relative to the total number of floret buds (abscised and intact) per inflorescence. Each value represents means of five replicates ± SE. Different letters indicate significant differences between treatments at each pH separately, at *P* ≤ 0.01 according to the Tukey–Kramer HSD test using one-way ANOVA. The pKa values of NAA and 2,4-D are 4.23 and 2.75, respectively.

The effect of the pH of the pulsing solution on the inhibitory effect of NAA and 2,4-D on floret bud abscission was determined at different time points after a 24-h pulsing treatment. When the pH of the NAA pulsing solution was equal to its pKa (4.23), about 60% of the floret buds already abscised at 2 days after pulsing, and the abscission rate reached 100% after 4 days (Figure 2A). Pulsing with 2,4-D at pH equal to its pKa (2.75) resulted in a similar abscission pattern to that obtained with NAA. Raising the pH of the NAA pulsing solution to 7.0 slightly reduced the abscission rate compared to that obtained at the pH equal to its pKa, particularly during the initial 3 days after pulsing (Figure 2B). In contrast, at pH 7.0, 2-4-D significantly reduced the abscission rate compared to that obtained at the pH equal to its pKa, and the inhibitory effect was significantly higher than that observed with NAA at all the time points. Only pulsing with NAA at pH 8.25 significantly increased its inhibitory effect on floret bud abscission at all the time points compared to that obtained at pH 7.0, but the inhibitory effect of 2,4-D at this pH was again higher than that obtained with NAA (Figure 2C). It is noteworthy, that neither of the buffers used to obtain the various pH levels had any effect on floret bud abscission (data not shown).

### Autoradiography of the Acropetal Transport of NAA and 2,4-D

**FIGURE 3:**
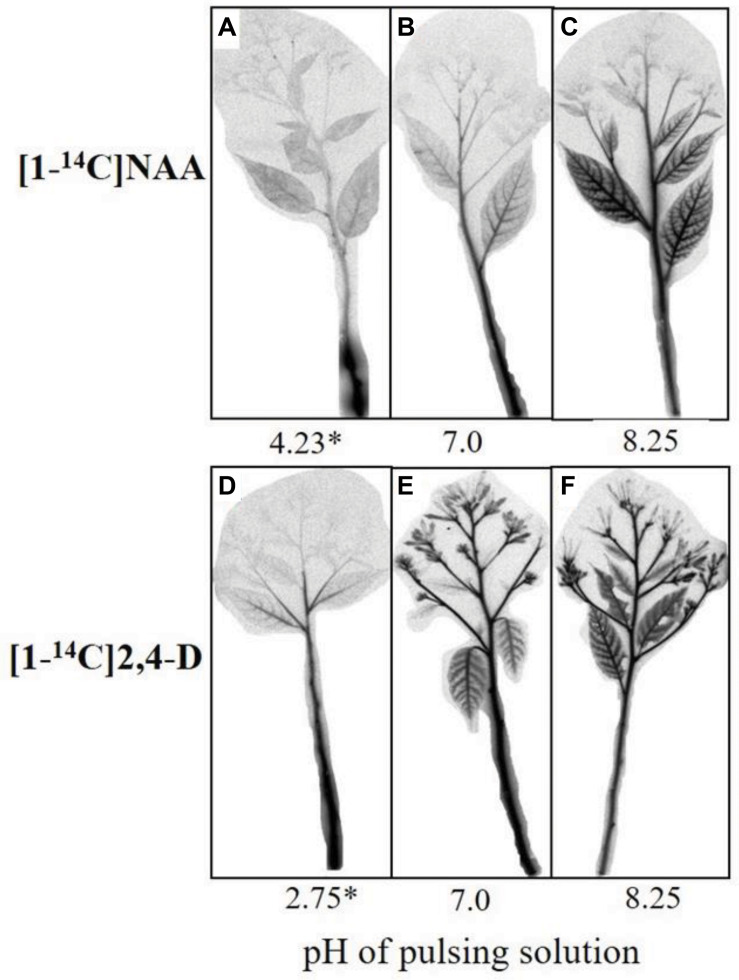
Autoradiography images of the acropetal transport of labeled NAA **(A–C)** or 2,4-D **(D–F)** in Red Cestrum cut flowers after pulsing at different pH levels. The cut flowers were pulsed for 24 h at 20°C with either 0.2 mM NAA or 2,4-D containing radiolabeled standards, at the indicated pH values. After pulsing the shoots were dried and exposed to a Fujifilm Imaging Plate for 48 h. Asterisk, the pH = pKa values of each auxin.

The effect of the pH of the pulsing solution on the acropetal transport of NAA and 2,4-D to the various organs of the cut flowers was evaluated by autoradiography conducted at the end of a 24-h pulsing. At the pH equal to the pKa, a strong label of NAA was observed in the lower shoot section, and only a trace of labeled NAA was detected in the upper shoot section and the lower leaves (Figure 3A). At pH 7.0, more NAA moved to the upper shoot section, and some label was also detected in the lateral shoots and particularly in the midribs of the lower leaves (Figure 3B). A much stronger label of NAA was observed in the above organs at pH 8.25, and a weak label was also detected at this pH in the rachises and the florets (Figure 3C). 2,4-D exhibited a different distribution pattern at the various pH levels than that obtained with NAA. Thus, some label of 2,4-D was already observed in the upper shoot section, lateral shoots, and midribs of the lower leaves at the pH equal to the pKa (Figure 3D). At pH 7.0 (Figure 3E) and particularly at pH 8.25 (Figure 3F), a strong label was detected in all organs of the cut flowers, including the sepals and florets of the open flowers.

### Effect of the pH on the Adsorption and/or Uptake of NAA and 2,4-D by Xylem Shoot Cells

**FIGURE 4:**
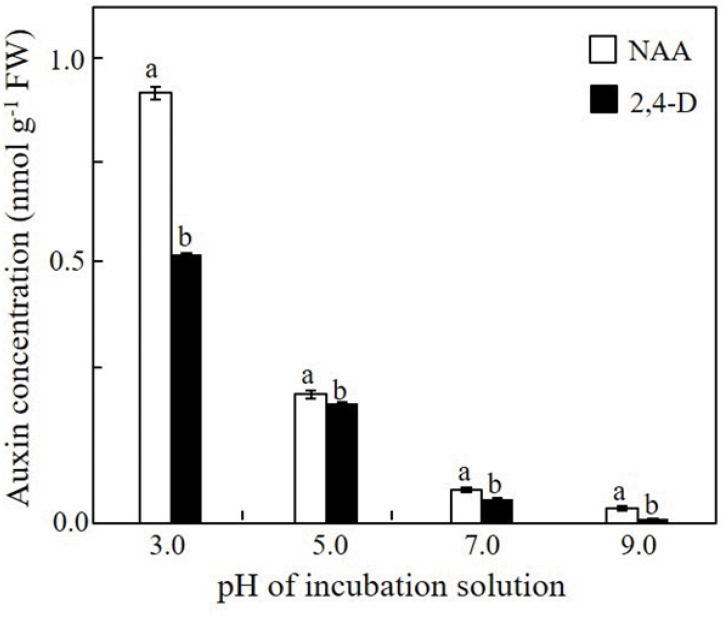
Effect of the pH of the incubation solution on the uptake/adsorption of NAA or 2,4-D by the xylem shoot sections excised from Red Cestrum cut flowers. Samples of 2- to 3-mm-long sections of xylem tissue were incubated for 1 h in 0.2 mM NAA or 2,4-D containing radiolabeled standards at the indicated pH. The extracted radiolabeled auxins served for determination of the auxin concentration in the treated xylem sections. Each value represents means of three replicates ± SE. Different letters indicate significant differences between treatments at each pH separately, at *P* ≤ 0.01 according to the Tukey–Kramer HSD test using one-way ANOVA.

The effect of the pH on the adsorption of NAA and 2,4-D onto the cell wall of the xylem shoot cells and/or on their uptake by these cells was determined immediately after a 1-h incubation at various pH levels. A gradual decrease in the adsorption and/or uptake of both auxins by the xylem shoot cells occurred with the increase in the pH of the pulsing solution from pH 3.0 to pH 9.0 (Figure 4). It should be noted that at all the pH levels, the amount of 2,4-D adsorbed and/or taken up by the xylem shoot cells was significantly lower than that of NAA.

### Expression of *CeIAA1* in the Floret AZ Following Pulsing With NAA and 2,4-D at Different pH Levels

**FIGURE 5:**
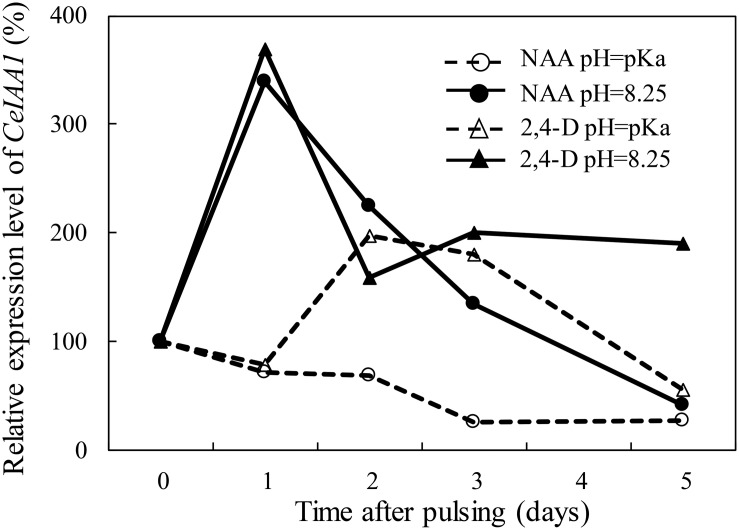
Effect of the pH of the pulsing solution on the influence of NAA and 2,4-D on the transcript level of *CeIAA1* gene in the floret AZ of Red Cestrum cut flowers during vase life. The cut flowers were pulsed for 4 h at 20°C and for additional 20 h at 4°C with 0.2 mM NAA or 2,4-D at pH = pKa or at pH = 8.25, and then placed in TOG-6 solution for further incubation under vase life conditions at 20°C. The qRT-PCR analysis was performed with RNA extracted from 2- to 3-mm-long floret AZ sections, excised at the indicated time points after pulsing during vase life. The expression level of *CeIAA1* was calculated relative to its basal level at the end of pulsing (0 time). The pKa values of NAA and 2,4-D are 4.23 and 2.75, respectively. The results represent average values of four biological replicates (two replicates of qRT-PCR reactions in each one of the two separate experiments performed).

In order to examine the uptake of NAA and 2,4-D by the floret bud AZ cells following pulsing of cut flowers with either one of these auxins at different pH levels, we evaluated the expression of *CeIAA1* at various time points after pulsing. This gene was one of the six *Aux/IAA* homologous genes cloned in the floret bud AZ of Red Cestrum cut flowers following auxin application, as reported in our previous study (Abebie et al., 2007). It was selected for the present study since it exhibited the highest increase in expression in response to applied NAA or 2,4-D, and it peaked 2 days after the initiation of the auxin treatments. The results demonstrate that at the pH equal to the pKa, the expression of *CeIAA1* in the floret bud AZ cells of the NAA-treated cut flowers remained low during the entire experimental period (Figure 5). The expression of *CeIAA1* in the floret bud AZ cells of the 2,4-D-treated cut flowers pulsed at pH equal to the pKa exhibited a different pattern. *CeIAA1* expression increased 2 days after pulsing, remained high on the third day, and decreased later on to the basal level. Raising the pH of the NAA pulsing solution from 4.23 (its pKa) to 8.25 increased by fourfold the expression of *CeIAA1* in the floret bud AZ cells one day after pulsing, and it gradually decreased later on to the basal level. Similar to NAA, *CeIAA1* expression increased considerably in the floret bud AZ cells of cut flowers pulsed with 2,4-D at pH 8.25, as compared to its pulsing at pH 2.75 (its pKa), reaching a peak of a fourfold increase 1 day after pulsing. However, unlike in NAA-treated flowers, *CeIAA1* expression in the 2,4-D-treated flowers at pH 8.25 decreased to a constant level, which remained about twofold higher than the basal level up to the end of the experiment. It should be noted that in our previous report we demonstrated that the expression of *CeIAA1* remained almost unchanged in the AZ of floret buds during vase life of the untreated cut flowers (Abebie et al., 2007).

### Effect of the pH on Physicochemical Properties of NAA and 2,4-D

The effect of the pH on the ionization rates and K_*OW*_ values of NAA and 2,4-D was studied in order to elucidate the effect of the pH on their adsorption onto cell wall components of xylem shoot cells and/or uptake by these cells. The data presented in Table 1 demonstrate that increasing the pH from 3.0 to 7.0 caused almost a complete ionization of the two auxins, with a significant concomitant decrease in their K_*OW*_ values. However, at the two lower pH levels, the ionization rates of 2,4-D were significantly higher than those of NAA, and its K_*OW*_ values were significantly lower.

**TABLE 1:** Effect of the pH on physicochemical properties of NAA and 2,4-D.

**Auxin _(pKa)_**	**Parameter**	**pH**			
		**3.0**	**5.0**	**7.0**	**9.0**
NAA_(__4_._23__)_	Percent ionization	5.60	86.00	99.80	99.99
	K_OW_	130.88 ± 5.09	6.93 ± 0.15	0.92 ± 0.01	0.07 ± 0.01
	log K_OW_	2.12	0.84	−0.03	−1.16
2,4-D_(__2_._75__)_	Percent ionization	64.00	99.00	99.80	99.99
	K_OW_	20.04 ± 1.80	2.04 ± 0.04	0.69 ± 0.02	0.25 ± 0.01
	log K_OW_	1.30	0.31	−0.16	−0.60

*Percent ionization was determined by using the Henderson–Hasselbalch equation. K_*OW*_ values were calculated from the equilibrium ratio of the auxin concentrations in the *n*-octanol and the aqueous phases. The results of K_*OW*_ represent means of four replicates ± SE.*

## Discussion

Previous studies of our group demonstrated that 2,4-D applied by the standard pulsing method (pH 3.4) had a higher acropetal transport capability, and hence a higher efficacy in inhibiting abscission of floret buds and florets in Red Cestrum cut flowers during vase life, than those of NAA (Abebie et al., 2005, 2006, 2007). Based on the common view on the factors which affect the acropetal transport capacity of week acids (Trapp, 2004; Kramer, 2006), it seemed quite reasonable that the differential acropetal transport capacity of the two auxins resulted from the difference in their pKa values. We hypothesized that because of this difference, raising the pH of the pulsing solution of Red Cestrum cut flowers would differentially affect the physicochemical properties of the two auxins. This could in turn affect their lipophilicity, which determines the rate of adsorption onto plant cell wall components and/or uptake by cells, and hence their acropetal transport capability. The data of the present research demonstrated that raising the pH of the pulsing solution of NAA and 2,4-D increased their transport capacity (Figure 3), resulting in a higher efficacy in inhibiting floret bud abscission (Figure 2). But as expected, the increase in 2,4-D transport capacity was significantly higher than that of NAA. Similarly, the pKa was reported to be an important factor in determining the phloem transport capability of acidic herbicides (Bromilow et al., 1990). The difference in the pKa of the two auxins was also demonstrated in their differential adsorption and/or uptake by xylem cells (Figure 4), which have a significant impact on the acropetal transport capability. It is noteworthy, that besides increasing the dissociation of the auxin molecules, which decreases their membrane permeability and adsorption onto cell wall components, the high pH can also inhibit the activity of 2,4-D influx carriers (see below), thus decreasing its ion trapping in cells adjacent to the vascular system.

Studying the effect of the pH on the physicochemical properties of NAA and 2,4-D indicates that a high pulsing solution pH led to a significant reduction in the log K_*OW*_, i.e., in the lipophilicity of both auxins (Table 1), so that the dissociated hydrophilic molecules failed to adsorb onto cell wall components and/or taken up by the cells. This leads in turn to their increased accumulation in the xylem, and hence to increased acropetal transport. A significant decline in the lipophilicity of weak acids with increasing pH was previously reported (Coupland, 1989). These data are in accordance with the observations showing the importance of the hydrophilic-lipophilic balance of xenobiotics for their transport capability in the vascular systems, for example in the phloem (Bromilow et al., 1990). Although both auxins exhibited a decrease in lipophilicity in response to the increase in the pH, the decrease in the lipophilicity of 2,4-D along the pH range studied was more significant than that of NAA, leading to a higher transport capability (Figure 3). It is noteworthy, that while the log K_*OW*_ of NAA at pH 3.0 (2.12) is comparable to the value reported in the literature (2.20), that of 2,4-D (1.30) was significantly lower than the reported value (2.90) (Jafvert et al., 1990). This could result from the fact that we determined the log K_*OW*_ of 2,4-D at a pH range higher than its pKa (2.75), in which more than 60% of its molecules are presumed to be dissociated (Table 1).

The relatively high lipophilicity of NAA led to its higher adsorption and/or uptake by the xylem cells compared to 2,4-D, particularly at the low pH level (Figure 4), indicating that lipophilicity of NAA is one of the major factors which hindered its acropetal movement compared to 2,4-D under the standard pulsing conditions (pH 3.4). Adsorption of lipophilic compounds onto plant material limits their movement (Barak et al., 1983; Trapp et al., 2001). For example, the more lipophilic herbicide trifluralin failed to move freely in the phloem because of its strong adsorption onto plant cells (Bromilow et al., 1990).

The data obtained in the present paper raise two questions. The first is related to the transport capability of NAA with the increase in the pH of the pulsing solution. The observations that the transport capability of NAA at pH 7.0, at which it was completely ionized (Table 1), was still limited (Figure 3), suggest that additional factors might affect its transport capability. One possible factor is the binding of NAA to apoplastic proteins, which reduces its level in the xylem. An example for these proteins is the Auxin Binding Protein 1 (ABP1). Although ABP1 is mostly localized in the endoplasmic reticulum (ER), there is evidence that a small fraction of it is localized in the apoplast, i.e., in the extracellular space between the plasma membrane and the cell wall (Feng and Kim, 2015; Grones and Friml, 2015). In Arabidopsis for example, Xu et al. (2014) reported that about 22% of ABP1 was secreted from the ER to the apoplast. There are reports showing that the apoplast pH (5.5) is more favorable for auxin binding to ABP1 than the ER pH (7.5) (Grones and Friml, 2015). Nevertheless, NAA is expected to bind to some extend to ABP1 also at pH 7.0. In addition, it was reported that ABP1 has a much higher affinity for NAA binding than for 2,4-D binding (Badescu and Napier, 2006). Various other auxin binding proteins might also differentially interfere with the acropetal transport of the two auxins. Riederer (2004) claimed that lipophilic substances, which are present along the transport pathway, might be an additional barrier to transport. If a molecule is not sufficiently hydrophilic, it could be partitioned into the lipophilic substances, thereby being trapped by them. Our data show that, based on the physicochemical characteristics of the two auxins, NAA is less hydrophilic than 2,4-D at pH 7.0 (Table 1), so that this process might interfere with its acropetal transport more than with 2,4-D transport.

The second question is related to the uptake of auxins at high pH levels. Theoretically, a high pH in the xylem is expected to inhibit the uptake of auxins by the target cells, which in the present study were the floret bud AZ cells. This however was not the case in this system, since the inhibitory effect of NAA and 2,4-D on floret bud abscission (Figure 2) and their induction of *CeIAA1* expression in the AZ cells (Figure 5) were considerably higher at pH 8.25 than those obtained at lower pH levels. In our previous study performed with Red Cestrum cut flowers, we cloned six *Aux/IAA* homologous genes, designated as *CeIAA1* to *CeIAA6*, in the floret bud AZ (Abebie et al., 2007). The *CeIAA1* gene was characterized as a late auxin-responsive gene, as its mRNA peaked in the floret bud AZ 2 days after the initiation of the NAA or 2,4-D pulsing treatments. These results suggest that this gene may also have a regulatory role in the abscission process of floret buds. Therefore, the highly expressed *CeIAA1* gene in the floret bud AZ in response to auxin treatments can serve as a marker of auxin activity in this system. It should be noted that increased expression of the *Aux/IAA* homologous genes in our previous study occurred only in response to NAA or 2,4-D treatments, and was positively correlated with the efficacy of these treatments to inhibit floret bud abscission.

Auxin uptake at the high pH used for pulsing might be explained by re-attainment of the normal pH (pH homeostasis) in the xylem following the termination of the pulsing treatment, as described in various plant systems (Savchenko and Heber, 2000; Geilfus, 2017). For example, Peters and Felle (1991) demonstrated that the apoplast pH of corn coleoptile segments changed quickly during incubation, reaching a constant level irrespective of the initial incubation medium pH. Another common example of the apoplastic pH regulation is related to auxin-induced growth (Hager, 2003). Auxin induces the activation of the plasma membrane-localized H^+^-ATPase, which leads to acidification of the apoplast, resulting in increased activity of cell wall-loosening enzymes, followed by cellular expansion by the turgor pressure. It is possible that the post pulsing TOG-6 disinfectant incubation solution (about pH 6.0) also contributed to the reduction of the xylem pH following pulsing at high pH levels, due to its relatively low pH. It is noteworthy that plant hormones, including auxins, can also move across cell membranes by means of transporters (Grones and Friml, 2015; Park et al., 2017). Delbarre et al. (1996) demonstrated that in suspension-cultured tobacco cells, uptake of NAA occurred mostly by diffusion, whereas that of 2,4-D occurred mostly by influx carriers. Similar to the penetration of auxins into cells by diffusion, the uptake of auxins by influx carriers is also dependent on the apoplast pH. Rubery (1978) reported that the pH optima of the carrier-mediated uptake of IAA and 2,4-D by suspension-cultured crown gall cells were pH 5.0 and 4.0, respectively. A later study approved the above data related to IAA uptake, by demonstrating that IAA binding to the Arabidopsis influx transporter AUX1 occurred in a pH-dependent manner, with maximum binding taking place between pH 5.0 and 6.0, and below and above this pH range the binding ability was very low (Carrier et al., 2008). The fact that none of the two auxin uptake processes, i.e., diffusion and influx carrier-mediated uptake, could operate at a high pH, supports our assumption that the xylem pH is adjusted to the normal one after pulsing, enabling the uptake of both auxins by the target cells.

The data of the present research indicate that the relatively high acropetal transport capability of 2,4-D is an important factor in determining its higher efficacy in inhibiting floret bud abscission compared to that of NAA. However, additional factors, namely a high auxin activity and a slow rate of metabolism in the plant tissues might contribute to the relatively high activity of 2,4-D (Enders and Strader, 2015; Peterson et al., 2016). 2,4-D was also demonstrated to be metabolized at a much slower rate than that of NAA in Red Cestrum cut flowers and leaf tissues (Abebie et al., 2005). However, the relatively high activity of 2,4-D might limit its use in various species due to phytotoxic effects. For example, pulsing of Red Cestrum cut flowers with 0.15 mM 2,4-D, which inhibited floret bud abscission, caused leaf yellowing (Meir et al., 1999). This was related to the increased ethylene production induced by 2,4-D, as the leaf yellowing was inhibited by STS, the ethylene action inhibitor. Similarly, Sacalis and Nichols (1980) reported that 2,4-D retarded petal senescence in carnation cut flowers, but caused damage to the vegetative tissues.

## Conclusion

From our data it can be concluded that the higher acropetal transport capability of 2,4-D compared to that of NAA results from its lower pKa value. Since the pKa affects the pH-dependent physicochemical properties of these two auxins, raising the pH of the pulsing solution improved significantly their acropetal transport, and contributed to their increased efficacy in reducing floret bud abscission of Red Cestrum cut flowers. Based on these results, increasing the pH can be practically used for pulsing application of weakly acidic compounds for agricultural purposes, such as improving the vase life of cut flowers. Considering the possible phytotoxic effects of 2,4-D, raising the pH of the auxin pulsing solution might be mainly important for increasing the efficacy of mild auxins such as NAA.

## Data Availability Statement

All datasets generated for this study are included in the article.

## Author Contributions

BA, SM, SP-H, JR, and RG were responsible for the conception, design of the experiments, and interpretation of the data. BA performed the laboratory experiments and the analyses of the data. MH assisted in the performance of the experiments. BA, JR, SM, and SP-H were involved in drafting the work, responsible for the writing, editing, and final approval of the version to be published. All authors revised and approved the final version.

## Conflict of Interest

The authors declare that the research was conducted in the absence of any commercial or financial relationships that could be construed as a potential conflict of interest.

## Footnotes

1 https://basicmedicalkey.com/physical-and-chemical-properties-of-drug-molecules/

## References

[B1] Abebie B. Goren R. Huberman M. Meir S. Philosoph-Hadas S. Riov J. (2005). Prevention of bud and floret abscission in *Cestrum* cut flowers is related to the mode of transport and metabolism of synthetic auxins. *Acta Hort.* 682 789–794. 10.17660/actahortic.2005.682.102

[B2] Abebie B. Lers A. Philosoph-Hadas S. Goren R. Riov J. Meir S. (2007). Differential effects of NAA and 2,4-D in reducing floret abscission in *Cestrum* (*Cestrum elegans*) cut flowers are associated with their differential activation of *Aux/IAA* homologous genes. *Ann. Bot.* 101 249–259. 10.1093/aob/mcm115 17591611 PMC2711013

[B3] Abebie B. Philosoph-Hadas S. Lers A. Goren R. Huberman M. Riov J. (2006). “The differential effectiveness of two synthetic auxins in delaying floret abscission in Red *Cestrum* cut flowers depends on their transport and metabolism,” in *Proceedings of the 33rd PGRSA Annual Meeting*, Sarasota, FL, 75–80.

[B4] Ascough G. D. Nogemane N. Mtshall N. P. van Staden J. (2005). Flower abscission: environmental control, internal regulation and physiological responses of plants. *South Afr. J. Bot.* 71 287–301. 10.1016/s0254-6299(15)30101-0

[B5] Ausubel F. M. Brent R. Kingston R. F. Moore D. D. Seidman J. G. Smith J. A. (1995). *Short Protocols in Molecular Biology*, 3rd Edn, New York, NY: John Wiley & Sons, Inc.

[B6] Badescu G. O. Napier R. M. (2006). Receptors for auxin: will it end in TIRs. *Trends Plant Sci.* 11 217–223. 16564202 10.1016/j.tplants.2006.03.001

[B7] Bagheri H. Hashemabadi D. Sedaghahhoor S. (2012). Improvement of vase life and postharvest quality of Alstroemeria hybrida flowers via naphthalene acetic acid (NAA). *Eur. J. Exp. Biol.* 2 2481–2484.

[B8] Ballantyne D. J. (1965). Senescence of daffodil (*Narcissus pseudonarcissus*) cut flowers treated with benzyladenine and auxin. *Nature* 205:819 10.1038/205819a0

[B9] Barak E. Dinoor A. Jacoby B. (1983). Adsorption of systemic fungicides and a herbicide by some components of plant tissues, in relation to some physicochemical properties of the pesticides. *Pestic. Sci.* 14 213–219. 10.1002/ps.2780140302

[B10] Bertosa B. Kojic-Prodic B. Wade R. C. Ramek M. Piperaki S. Tsantili-Kakoulidou A. (2003). A new approach to predict the biological activity of molecules based on similarity of their interaction fields and the log P and log D values: application to auxins. *J. Chem. Info. Comp. Sci.* 43 1532–1541. 10.1021/ci034063n 14502487

[B11] Briggs G. G. Rigitano R. L. O. Bromilow R. H. (1987). Physicochemical factors affecting uptake by roots and translocation to shoots of weak acids in barley. *Pestic. Sci.* 19 101–112. 10.1002/ps.2780190203

[B12] Bromilow R. H. Chamberlain K. Evans A. A. (1990). Physicochemical aspects of phloem translocation of herbicides. *Weed Sci.* 38 305–314. 10.1017/s0043174500056575

[B13] Carrier D. J. Abu Baker N. T. Swarup R. Callaghan R. Napier R. M. Bennett M. J. (2008). The binding of auxin to the *Arabidopsis influx* transporter AUX1. *Plant Physiol.* 148 529–535. 10.1104/pp.108.122044 18614710 PMC2528085

[B14] Chamberlain K. Evans A. A. Bromilow R. H. (1996). 1-Octanol/water partition coefficient (KOW) and pKa for ionisable pesticides measured by a pH-metric method. *Pestic. Sci.* 47 265–271. 10.1002/(sici)1096-9063(199607)47:3<265::aid-ps416>3.0.co;2-f

[B15] Coupland D. (1989). “Factors affecting the phloem translocation of foliage-applied herbicides,” in *Mechanism and Regulation of Transport Processes*, eds Atkin R. K. Clifford D. R. (Berlin: Springer), 85–112.

[B16] Delbarre A. Muller P. Imhoff V. Guern J. (1996). Comparison of mechanisms controlling uptake and accumulation of 2,4-dichlorophenoxy acetic acid, naphthalene-1-acetic acid, and indole-3-acetic acid in suspension-cultured tobacco cells. *Planta* 198 532–541. 10.1007/bf00262639 28321663

[B17] Diaz-Sala C. Hutchison K. W. Goldfarb B. Greenwood M. S. (1996). Maturation-related loss in rooting competence by loblolly pine stem cuttings: the role of auxin transport, metabolism and tissue sensitivity. *Physiol. Plant.* 97 481–490. 10.1034/j.1399-3054.1996.970310.x 11841302

[B18] Enders T. A. Strader L. C. (2015). Auxin activity: past, present, and future. *Am. J. Bot.* 102 180–196. 10.3732/ajb.1400285 25667071 PMC4854432

[B19] Feng M. Kim J.-Y. (2015). Revisiting apoplastic auxin signaling mediated by AUXIN BINDING PROTEIN 1. *Mol. Cells* 38 829–835. 10.14348/molcells.2015.0205 26467289 PMC4625063

[B20] Geilfus C.-M. (2017). The pH of the apoplast: dyanamic factor with functional impact under stress. *Mol. Plant* 10 1371–1386. 10.1016/j.molp.2017.09.018 28987886

[B21] Grimm E. Neumann S. Jacob F. (1986). Transport of xenobiotics in higher plants. III. Absorption of 2,4-D and 2,4-dichloroanisole by isolated conducting tissue of Cyclamen. *Biochem. Physiol. Pflanzen.* 181 69–82.

[B22] Grones P. Friml J. (2015). Auxin transporters and binding proteins at a glance. *J. Cell Sci.* 128 1–7. 10.1242/jcs.159418 25556248

[B23] Hager A. (2003). Role of plasma membrane H+-ATPase in auxin-induced elongation of growth: Historical and new aspects. *J. Plant Res.* 116 483–505. 10.1007/s10265-003-0110-x 12937999

[B24] Huberman M. Zehavi U. Stein W. D. Etxeberria E. Goren R. (2005). In vitro sugar uptake by grapefruit (*Citrus paradisi*) juice-sac cells. *Func. Plant Biol.* 32 357–366. 10.1071/FP04125 32689137

[B25] Jafvert C. T. Westall J. C. Grieder E. Schwarzenbach R. P. (1990). Distribution of hydrophobic ionogenic organic compounds between octanol and water: organic acids. *Environ. Sci. Technol.* 24 1795–1803. 10.1021/es00082a002

[B26] Kerler F. Schönherr J. (1988). Accumulation of lipophilic chemicals in plant cuticles: prediction from octanol/water partition coefficients. *Arch. Environ. Contam. Toxicol.* 17 1–6. 10.1007/bf01055146

[B27] Kramer E. M. (2006). How far can a molecule of weak acid travel in the apoplast or the xylem? *Plant Physiol.* 141 1233–1236. 10.1104/pp.106.083790 16896235 PMC1533949

[B28] Leo A. (2000). “Octanol/water partition coefficients,” in *Handbook of Property Estimation Methods for Chemicals*, eds Boethling R. S. Mackay D. (Boca Raton, FL: Lewis Publishers), 89–114.

[B29] Leo A. Hansch C. Elkins D. (1971). Partition coefficients and their uses. *Chem. Rev.* 71 525–616. 10.1021/cr60274a001

[B30] Liao Z. H. Chen M. Guo L. Gong Y. F. Tang F. Sun X. F. (2004). Rapid isolation of high quality total RNA from taxus and ginkgo. *Prep. Biochem. Biotech.* 34 209–214. 10.1081/pb-200026790 15461137

[B31] Ludwig-Müller J. Raisig A. Hilgenberg W. (1995). Uptake and transport of indole-3-butyric acid in *Arabidopsis thaliana*: comparison with other natural and synthetic auxin. *J. Plant Physiol.* 147 351–354. 10.1016/s0176-1617(11)82166-8

[B32] Meir S. Philosoph-Hadas S. Salim S. Davidson H. Tamari Y. Gutman S. (1999). Prevention of floret abscission in Cestrum cut flowers by pulsing treatments with synthetic chlorophenoxy auxins and STS. *Bulletin of Israeli Flower Growers* 4, 83–89. In Hebrew.

[B33] Meir S. Sundaresan S. Riov J. Agarwal I. Philosoph-Hadas S. (2015). Role of auxin depletion in abscission control. *Stewart Postharvest. Rev.* 11 1–15. 10.2212/spr.2015.2.2 25112557

[B34] OECD (1987). *OECD Guidelines for the Testing of Chemicals, Section 4.* Paris: OECD Publishing.

[B35] Park J. Lee Y. Martinoia E. Geisler M. (2017). Plant hormone transporters: What we know and what we would like to know? *BMC Biol.* 15:93. 10.1186/s12915-017-0443-x 29070024 PMC5655956

[B36] Peters W. S. Felle H. (1991). Control of apoplast pH in corn coleoptile segments. I: the endogenous regulation of cell wall pH. *J. Plant Physiol.* 137 655–661. 10.1016/s0176-1617(11)81217-4

[B37] Peterson M. A. McMaster S. A. Riechers D. E. Skelton J. Stahlman P. W. (2016). 2,4-D: past, present, and future: a review. *Weed Technol.* 30 303–345.

[B38] Petrášek J. Friml J. (2009). Auxin transport routes in plant development. *Development* 136 2675–2688. 10.1242/dev.030353 19633168

[B39] Popp C. Burghardt M. Friedmann A. Riederer M. (2005). Characterization of hydrophilic and lipophilic pathways of *Hedera helix* L. cuticular membranes: permeation of water and uncharged organic compounds. *J. Exp. Bot.* 56 2797–2806. 10.1093/jxb/eri272 16143718

[B40] Reid M. S. Jiang C.-Z. (2012). Postharvest biology and technology of cut flowers and potted plants. *Hort. Rev.* 40 1–54. 10.1002/9781118351871.ch1

[B41] Riederer M. (2004). “Uptake and Transport of *Xenobiotics*,” in *Plant Toxicology*, 4th Edn, eds Hook B. Elstner E. F. (Boca Raton: CRC Press), 131–150. 10.1201/9780203023884

[B42] Rigitano R. L. O. Bromillow R. H. Briggs G. G. Chamberlain K. (1987). Phloem translocation of weak acids in *Ricinus communis*. *Pestic. Sci.* 19 113–133.

[B43] Rubery P. H. (1978). Hydrogen ion dependence of carrier-mediated auxin uptake by suspension-cultured crown gall cells. *Planta* 142 203–206. 10.1007/bf00388213 24408103

[B44] Sacalis J. N. Nichols R. (1980). Effect of 2,4-D uptake on petal senescence in cut carnation flowers. *Hortscience* 15 499–500.

[B45] Savchenko G. Heber V. (2000). pH regulation in apoplastic and cytoplastic cell compartments of leaves. *Planta* 211 246–255. 10.1007/s004250000280 10945219

[B46] Shimizu-Yumoto H. Ichimura K. (2010a). Combination pulse treatment of 1-naphthaleneacetic acid and aminoehoxyvinyl glycine greatly improves postharvest life in cut Eustoma flowers. *Postharvest. Biol. Technol.* 56 104–107. 10.1016/j.postharvbio.2009.10.001

[B47] Shimizu-Yumoto H. Ichimura K. (2010b). Postharvest physiology and technology of cut Eustoma flowers. *J. Jpn. Soc. Hort. Sci.* 79 227–238. 10.2503/jjshs1.79.227

[B48] Sterling T. M. (1994). Mechanisms of herbicide absorption across plant membranes and accumulation in plant cells. *Weed Sci.* 42 263–276. 10.1017/s0043174500080383

[B49] Trapp S. (2004). Plant uptake and transport models for neutral and ionic chemicals. *Environ. Sci. Pollut. Res.* 11 33–39. 10.1065/espr2003.08.169 15005138

[B50] Trapp S. Miglioranza K. S. B. Mosbaek H. (2001). Sorption of lipophilic organic compounds to wood and implications for their environmental fate. *Environ. Sci. Technol.* 35 1561–1566. 10.1021/es000204f 11329702

[B51] Weisman Z. Riov J. Epstein E. (1988). Comparison of movement and metabolism of indole-3-acetic acid and indole-3-butyric acid in mung bean cuttings. *Physiol. Plant.* 74 556–560. 10.1111/j.1399-3054.1988.tb02018.x

[B52] Wojciechowska N. Sobieszczuk-Nowicka E. Bagniewska-Zadworna A. (2018). Plant organ senescence – regulation by manifold pathways. *Plant Biol.* 20 167–181. 10.1111/plb.12672 29178615

[B53] Xu T. Dai N. Chen J. Nagawa S. Cao M. Li H. (2014). Cell surface ABP1-TMK auxin-sensing complex activates ROP GTPase signaling. *Science* 343 1025–1028. 10.1126/science.1245125 24578577 PMC4166562

